# Approach to Neurometabolic Diseases from a Pediatric Neurological Point of View

**Published:** 2015

**Authors:** Parvaneh KARIMZADEH

**Affiliations:** 1 Department of Pediatric Neurology, Pediatric Neurology Research Center, Shahid Beheshti University of Medical Sciences, Tehran/Iran; 2 Pediatric Neurology Center of Excellence, Department of Pediatric Neurology, Mofid Children Hospital, Faculty of Medicine, Shahid Beheshti University of Medical Sciences, Tehran, Iran

**Keywords:** Neurometabolic disorders, Neurological manifestation, Electroencephalography, Early detection, Early treatment

## Abstract

**Objective**

Neurometabolic disorders are an important group of diseases that mostly are presented in newborns and infants.

Neurological manifestations are the prominent signs and symptoms in this group of diseases. Seizures are a common sign and are often refractory to antiepileptic drugs in untreated neurometabolic patients.

The onset of symptoms for neurometabolic disorders appears after an interval of normal or near normal growth and development.Additionally, affected children may fare well until a catabolic crisis occurs.

Patients with neurometabolic disorders during metabolic decompensation have severe clinical presentation, which include poor feeding, vomiting, lethargy, seizures, and loss of consciousness.

This symptom is often fatal but severe neurological insult and regression in neurodevelopmental milestones can result as a prominent sign in patients who survived.

Acute symptoms should be immediately treated regardless of the cause.

A number of patients with neurometabolic disorders respond favorably and, in some instances, dramatically respond to treatment.

Early detection and early intervention is invaluable in some patients to prevent catabolism and normal or near normal neurodevelopmental milestones.

This paper discusses neurometabolic disorders, approaches to this group of diseases (from the view of a pediatric neurologist), clinical and neurological manifestations, neuroimaging and electroencephalography findings, early detection, and early treatment.

## Introduction

Neurometabolic diseases refer to a group of disorders that are characterized by a lack or dysfunction of an enzyme or vitamin necessary for a specific chemical reaction in the body. 

A lack or dysfunction of an enzyme can cause a deficiency of an essential product (metabolites) in the brain that leads to specific types of neurometabolic diseases. A lack of these essential metabolites may impair normal brain development. Further, this lack of essential metabolites can also cause stored materials that may become toxic to the brain especially in the developing brains in children. 

The toxic material would otherwise be metabolized by the specific enzymes. Accumulation of these toxic metabolites has harmful effect on brain and lead to loss of nerve cells and breakdown of brain white and gray matters.

Neurometabolic diseases are inherited disorders that are individually rare but as a group have a significant burden.

Defects or mutations in a single gene disrupt structure and function of corresponding protein enzyme that lead to significant neurometabolic disease. Although children may have the same gene mutation but without suffering the same neurometabolic problems, some children are severely ill at birth and die soon after presentation of symptoms while others might be only mildly affected and their symptoms present later in life.

The most common presenting signs of inborn errors of metabolism are neurological manifestations.

Global neurodevelopmental delay is only the first symptoms in many of these progressive disorders.

**Signs and Symptoms**

The presenting symptoms for neurometabolic diseases are caused by progressive destruction of mental, motor, and perceptual functions. The symptoms can appear at any age from newborns into adulthood.

In some neurometabolic disorders, the structure of brain has abnormally developed before birth (such as in Zellweger syndrome), but in other disorders just after birth and at the start of feeding, symptoms become apparent. The symptoms include seizures, loss of consciousness, hypotonia, poor feeding, and respiratory distress,among others.

Other neurometabolic disorders present in young infants with neurodevelopmental delay. These infants fail to progress to normal development and suffer recurrent episodes of vomiting, lethargy, and loss of consciousness brought on by environmental stresses such as upper respiratory tract infections, vaccination, or surgery, among others. (1).

An abnormal odor of the body and urine, microcephaly or macrocephaly, visual loss and auditory loss, and seizures in these patients may lead to further investigations of neurometabolic diseases.

After one or two years, children may show regressions in skills, i.e. learned motor and mental skills (2).

Older infants may also show hepatosplenomegaly, coarse facies, skeletal abnormalities, and uncontrolled seizures. Sometimes parents report abnormalities with the hair and skin, especially dermatitis in sun-exposed regions.

Children may show an abnormal gait as with ataxia, mental decline, abnormal behavior, and sleep disorders.

Neurological examinations may reveal abnormal tones, spasticity, or hypotonicity, and brisk deep tendon reflexes.

In addition, children may show visual loss, poor attention, speech disturbance, and cerebellar signs.

In adulthood, neurometabolic diseases mainly have psychiatric sign and aggressiveness, mood disorders, and behavioral disorders are the major sign of neurometabolic disorders.

**Disorders of Amino acids**

**Phenylketonuria (PKU)**

PKU is caused by phenylalanine hydroxylase deficiency, a hepatic enzyme that converts phenylalanine to tyrosine (two important aminoacids).

Phenylalanine hydroxylase deficiency causes an accumulation of phenylalanine, which is then converted to phenyl pyruvic acid and phenyl lactic acid. These metabolites are excreted in the urine.

Phenylalanine and their metabolites are neurotoxic and untreated patients are typically profoundly mentally retarded.

Children with PKU appear normal at birth and have normal neurodevelopmental milestones in early infancy, but gradually they show reduced head circumference and developmental delays. Children with PKU may have a musty odor especially in the urine due to metabolites of phenyl lactic acid and phenyl pyruvic acid. Behavioral disorders and autistic spectrum disorders are common in these children.

Brain Magnetic resonance imaging (MRI) shows a high intensity in periventricular white matter (dysmyelination). These findings are reversible with a restricted diet of phenylalanine.

Abnormal findings in the white matter demonstrate restricted diffusion of water which indicates high myelin turnovers.

**Fig 1:**
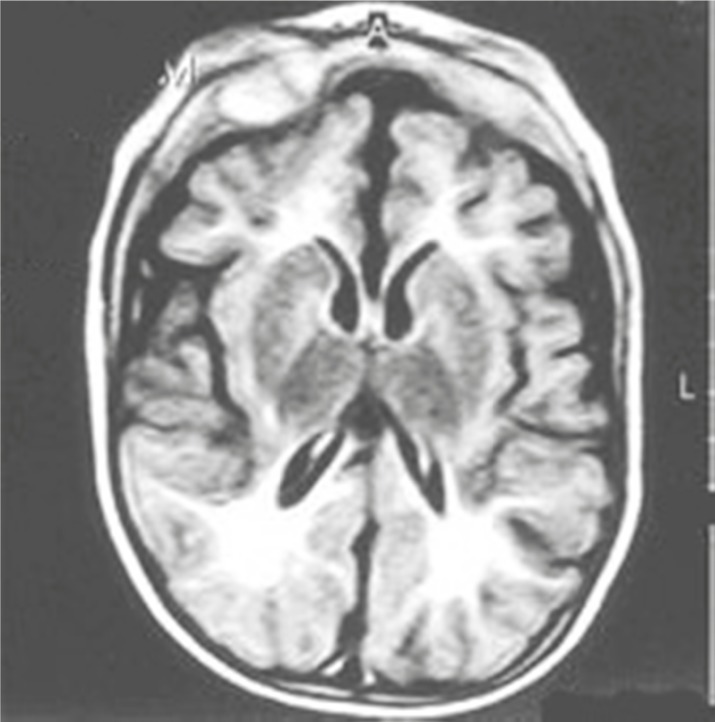
Abnormally high signal intensity in white matter regions around the anterior and posterior horns of both lateral ventricles and brain atrophy in phenylketonuria

The severity of MRI findings (white matter involvement) correlate with mean phenylalanine levels in the past years and point to phenylalanine levels at the time of brain imaging (Figure 1) (3).

Many studies have revealed correlation between phenylalanine serum levels and MRI white matter involvement in conventional methods and a higher sensitivity in Diffusion Weighted Imaiging (DWI).

Karimzadeh et al in the study of “MRI Changes in 30 patients with phenylketonuria” indicated an assessment of mean 1-year phenylalanine levels are the best indicator of white matter involvement and are more related with MRI scores compared to phenylalanine levels at the time of imaging (4).

This study suggests the use of brain MRIs and evaluation of white matter involvement to monitor long-term control of phenylalanine levels in PKU cases.

In order to prevent neurotoxicity from phenylalanine, patients with PKU need a diet that restricts phenylalanine. 

This restricted diet causes normal plasma phenylalanine and stops the excretion of phenylpyruvic acid and phenyl lactic acid. The selective restrictions of Phe. and tyrosine supplementation lead to normal growth and neurodevelopment.

An abnormal EEG that includes generalized paroxysmal activity and a generalized slowing are common even in patients who have received treatments. 

High serum phenylalanine levels in untreated women with PKU can cross the placenta and cause Microcephaly(70%), intrauterine growth retardation (40%), and congenital heart disease (12%) in the fetus.

Mothers with PKU should be monitored carefully with a restricted diet of phenylalanine and the diet should be started 3 months before conception.

**Maple Syrup Urine Disease**

At first, Menkes et al described this disease. Maple Syrup Urine Disease (MSUD) is caused by a mitochondrial branched chain α-ketoacid dehydrogenase deficiency. 

The defect of this enzyme leads to accumulation of branched chain amino acids and branched chain α-ketoacids.

MSUD has 5 types: classic, intermediate, intermittent, thiamine responsive, and dihydrolipoyl dehydrogenase deficiency.

The clinical manifestations are different and depend on the level of enzyme activity.

In the classic type of MSUD, with severe encephalopathy, symptoms start in newborns and early infancy and include poor feeding, hypertonia, opisthotonic posturing, failure to thrive, respiratory disorders, seizures, and urine that smells like maple syrup.

Affected infants may be misdiagnosed with sepsis and die from seizures and coma.

Hyponatremia and cerebral edema are common during acute metabolic states. Infants who survive ametabolic crisis have severe neurodevelopmental delays as well as visual and mental deficits. 

**Fig 2:**
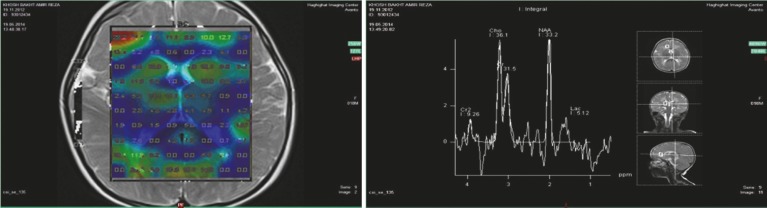
Single proton MR spectroscopy shows the presence of branched-chain amino acids resonating at 0.9–1.0 ppm

A brain MRI is typically abnormal in MSUD patients. Magnetic resonance spectroscopy (MRS) shows a peak of branched chain amino acids and branched chain α-ketoacids resonating at 0.9–1.0 ppm, especially during a metabolic crisis (Figure 2).

**Organic-acidemia**

Ketotic hyperglycinemia includes propionic-academia, methylmalonic-academia, and isovaleric-academia all have similar clinical pictures.

A severe metabolic crisis presents as hyperammonemia, severe ketoacidosis, vomiting, poor feeding, lethargy, and coma. Undiagnosed children often die in early infancy. The clinical presentation depends on enzymatic deficiencies. Even in patients with mild clinical presentations, in late infancy and childhood the metabolic crisis can be fatal. During acute metabolic crisis, treatment is limited to catabolism and restricted protein intake.

Propionic-acidemia is a congenital neurometabolic disorder of organic acid metabolism, which is recessively inherited, characterized by a spectrum of clinical, and biochemical manifestations.

This disorder can present with life-threatening ketoacidosis, lethargy, failure to thrive, and developmental delays.

Karimzadeh et al reported that for 10 patients with propionic-acidemia, the first and chief complaint in 70% of patients were neurological disorders, such as developmental delay and seizures. In this study, 70% of refractory seizures were controlled with antiepileptic drug and L-carnitine (5).

**Fig 3:**
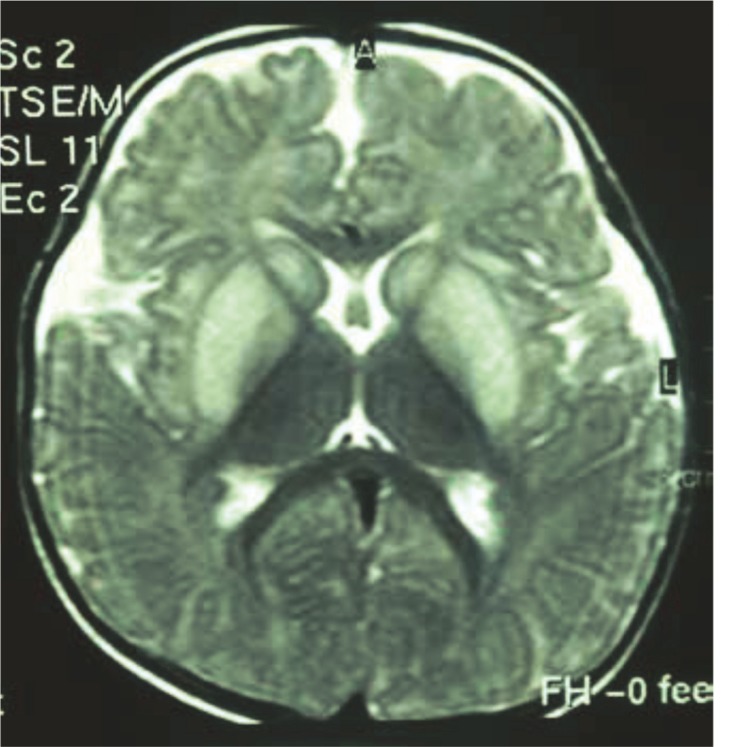
Abnormal Signal in Bilateral Basal Ganglia and Brain Atrophy in an MRI from a 4-year-old boy with propionic-acidemia

MRIs in patients with organic-academia show a pathology of the bilateral basal ganglia and some abnormal nonspecific intensity in the periventricular white matter (especially during an acute metabolic state). Children with severe handicaps and global neurodevelopmental delays show severe cerebral atrophy in cranial MRIs (Figure 3).

Previous studies, Ozand and Karimzadeh et al, have shown that early intervention in propionic-acidemia caused partial improvements in the demyelination of white matter but basal ganglia involvement did not completely resolve (5,6).

Methylmalonic-acidemia, another inborn error of amino acid metabolism, presents with neurologic deficits, metabolic acidosis, vomiting, lethargy, anorexia, and severe ketoacidosis. 

Karimzadeh et al indicated that in a study with 20 patients who have methylmalonic-acidemia, 60% had a history of seizures (30% tonic, 20% GTC, and 10% infantile spasm); 20% had refractory seizures; all types of refractory seizures were controlled after diagnosis and treatment of methylmalonic-academia (7).

In this study, MRIs from 50% of patients had brain atrophy, 15% had basal ganglia involvement (mostly in putamen), 10% had abnormal signal in periventricular white matter regions,and 30% had a normal brain MRI (7).

Radmanesh et al indicated that 52 patients with methylmalonic-academia, their cortical atrophy, and ventricular dilation were observed from neuroimaging (8).

Biotinidase deficiency is a neurometabolic disorders that patients respond to high doses of oral Biotin very well. 

Symptoms include alopecia, skin rash, neurodevelopmental delay, and seizures as well as visual and hearing impairments.

**Fig 4:**
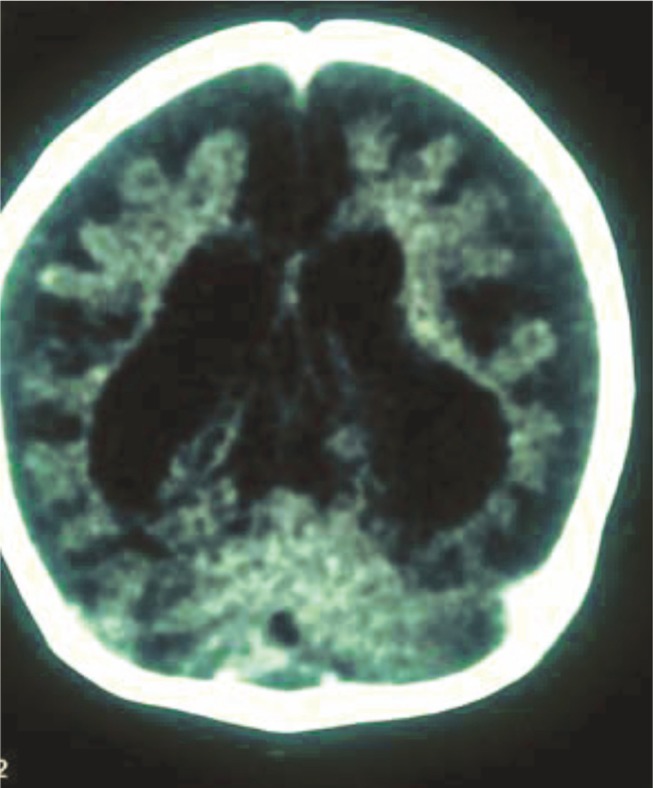
Severe brain atrophy in a patient with biotinidase deficiency

A cranial MRI shows abnormal signal intensity in the periventricular white matter, which is extensive in some patients (Figure 4).

One study indicated that16 patients with biotinidase deficiency showed the following:75% of patients had abnormal neuroimaging and of these, 56% had generalized brain atrophy and delay myelination.

Additionally, this study showed seizures and skin manifestations were improved after biotin therapy (9).

Karimzadeh et al study on Glutaricaciduria type 1 and with a 5-year follow up of these patients indicated no pattern of macrocephaly at birth.However, 25% of these patients had a larger head circumference than weight, but further follow-ups showed that 50% of these patients had macrocephaly (greater than 95%). The 5-year follow-up showed that patients with early diagnosis had a more favorable clinical response to treatment (10).

In this study, serial neuroimaging were done in seven patients and five of them (71%) followed a particular pattern indicated by fronto temporal atrophy and a widening of the Sylvian sulcus with white matter and basal ganglia involvement and subependymal cyst (10). 

**Disorders of Carbohydrate Metabolism**

Galactosemia is an inborn error of carbohydrate metabolism (deficiency of Galactose 1-phosphate uridyl transferase) that causes neurologic manifestations that include impaired mental development, vomiting, cataracts, pseudotumor cerebri, and cerebral edema as well as other signs such as hepatomegaly and failure to thrive, among others.

Clinical manifestations usually appear after initiation of milk feeding and increase in severity in the first days and months of life.

Impaired mental development is an important manifestation and is more severe in those who are not diagnosed as a newborn or in early infancy.It is common for untreated patients to be hyperactive and have impaired mental development. A relationship between mental decline and compliance with dietary restrictions was evident in average IQ, especially as regards poorly compliant patients who had an IQ below 70.

**Mitochondrial disorders**

The body’s nucleated cells contain 500–2000 mitochondria. Some organs such as the brain, heart, and skeletal muscles are highly energy dependent. 

Therefore, these organs are vulnerable to defects with energy metabolism.

In the cone cell photoreceptors of the eye, mitochondria make up 80% of the intracellular volume meanwhile in extraocular muscles like the lateral rectus, mitochondria occupies 60% of intracellular volume and 40% for the heart muscles.

In the liver, mitochondria are specialized to detoxify ammonia in the urea cycle and are required for neurotransmitter metabolism.

Mitochondrial encephalomyopathies are genetically, biochemically, and clinically heterogeneous group of disorders associated with abnormalities of oxidative phosphorylation.

There is no absolute gold standard to establish a diagnosis of mitochondrial encephalomyopathy. Lethal infantile mitochondrial disease is characterized by a fulminant neonatal onset (death within 6 months of life) cardiomyopathy,and myopathy.

Patients with Sengers syndrome are diagnosed with congenital cataracts, hypertrophic cardiomyopathy, mitochondrial myopathy, and lactic acidosis. 

Another syndrome as Barth syndrome, which is diagnosed with left ventricular non compaction (LVNC), skeletal myopathy, 3-methylglutaconic aciduria, and short stature.

Diagnostic criteria for pediatric mitochondrial disorders have been modified from the adult classification system known as “The Modified Walker Criteria” (Table1).

The Modified Walker Criteria is useful to assess the frequency of major clinical manifestations in a population of infants and children with a definite diagnosis of mitochondrial disease because the natural history of this heterogeneous group of disorders remains largely unknown.From this, we are able to gather a large group of pediatric patients with a definite diagnosis of a mitochondrial disorder and study the clinical histories (11, 12).

Neurological manifestations are important signs for these disorders. Most neurological manifestations include the following: muscle weakness (proximal muscle more affected than distal and upper extremities are more affected than lower extremities), hypotonia, peripheral neuropathy, ataxia, ptosis, ophthalmoplegia, bulbar signs, spasticity stroke-like episodes, migraine, headaches, tremors, chorea, ballismus, dystonia, seizures and myoclonus.

Mitochondrial disorders can present with some classic mitochondrial syndromes such as chronic progressive external ophthalmoplegia (CPEO), Leber hereditary optic neuropathy (LHON), mitochondrial encephalopathy, lactic acidosis, and stroke like syndromes (MELAS), myoclonic epilepsy with ragged red fibers (MERRF), mitochondrial myopathy (MNGIE), peripheral neuropathy, gastrointestinal encephalopathy, neuropathy, ataxia, retinitis pigmentosa (NARP), and Kearns-Sayre syndrome are the most important (13,14).

Mitochondrial DNA depletion syndrome, also known as Alpers syndrome, is an autosomal recessive disorder characterized by clinical findings of psychomotor retardation, intractable seizure, and liver failure. Seizures may initially be focal and then generalize tonic clonic seizure arethe prominent signs.

Epilepsia partialis continua and convulsive status epilepticus are common. The disorder, diagnosed in infants and young children, is progressive and often leads to death from hepatic failure or status epilepticus before age 3. 

**Table 1:** Modified Walker Criteria Applied to Children of Mitochondrial Disease 2 major criteria or 1 major plus 2 minor = Mitochondrial Disorders (11)

	**Major Criteria**	**Minor Criteria**
**Clinical**	Clinically complete Respiratory chain encephalomyopathy or a mitochondrial cytopathy defined as fulfilling all 3 of the following	Symptoms compatible with a Respiratory Chain defect
**Histology**	>2% ragged red fibers in skeletal muscle	Smaller numbers of RRF, Sub sarcolemmal Accumulation of Mitochondria, or widespread electron microscopy abnormalities of mitochondria
**Enzymology**	Cytochrome c oxidase negative fibers or residual activity of a RC complex <20% in a tissue; <30% in a cell line, or <30% in 2 or more tissues	Antibody-based demonstration of an RC defect or residual activity of an RC complex 20%–30% in a tissue, 30%–40% in a cell line, or 30%–40% in 2 or more tissues
**Functional**	Fibroblast ATP synthesis rates >3 SD below mean	Fibroblast ATP synthesis rates 2–3 SD below mean, or fibroblasts unable to grow in galactose media
**Molecular**	Nuclear or mtDNA mutation of undisputed pathogenicity	Nuclear or mtDNA mutation of probable pathogenicity
**Metabolic**		One or more metabolic indicators of impaired metabolic function

The clinical manifestations in mitochondrial disorders are wide. Conditions are divided into the following groups:

**Conditions with Nuclear DNA Defects**

1. Substrate transport defect

2. Carnitine transporter deficiency

3. Carnitine palmitoyltransferase I deficiency

4. Carnitine–acylcarnitine translocase deficiency

5. Carnitine palmitoyltransferase II

6. Defects of Substrate Utilization

7. Pyruvate carboxylase deficiency,

8. Pyruvate dehydrogenase complex deficiency

9. Defects of beta-oxidation

10. Defects of oxidation–phosphorylationcoupling

11. Defects of the Krebs cycle

12.Defects of the respiratory chain

13. Defects of protein importation

14. Defects of the lipid milieu of the inner mitochondrial membrane

15.Defects of mitochondrial motility, fusion, and fission

**Inherited Conditions Associated with Mitochondrial DNA Defects**

1.Point mutations defects

2.Protein-coding genes

3.Point mutations affecting synthetic genes

4.Acquired conditions associated with mitochondrial

dysfunction

In mitochondrial disorders, any organ can be involved.

**Peroxisomal disorders**

Peroxisomal disorders are a group of heterogeneous neurometabolic diseases that show the dysfunction of peroxisome.

**Adrenoleukodystrophy**

X-linked adrenoleukodystrophy(X-linked ALD) is an inherited, recessive, neurodegenerative disease that affects the white matter of the brain and the adrenal gland.

The disorder is caused by mutations in the ABCD1gene and leads to a defect in the peroxisomal transport membrane protein (ABCD1),which impairs peroxisomal b-oxidation, and then,results in the accumulation of very long-chain fatty acids. It is a progressive disorder characterized by Addison’s disease, dementia, and neurological deterioration (15,16).

There are several distinct clinical phenotypes that range from cerebral forms, adrenomyeloneuropathy (AMN) to asymptomatic persons or isolated adrenal insufficiency without CNS involvement, i.e. Addison’s disease only. 

Childhood cerebral ALD and adult adrenomyeloneuropathy are common clinical phenotypes.

Cerebral X-ALD can be presented as childhood, adolescent, and adult forms.

Affected boys with the childhood type usually present before 10 years of age (typically between 4–8 years of age) characterized as a rapidly progressive disorder with ataxia, spasticity, deafness, visual deficits, personality changes, and seizures. The less common adolescent form after 10 years of age demonstrates a similar course. Cerebral X-ALD is frequently associated with Addison’s disease, but primary adrenal insufficiency may coexist or develop after neurological disturbances. 

Typical MR findings in patients with cerebral ALD are well documented and consist of bilateral white matter abnormalities. Typically, they occur initially in the posterior cerebral regions and progress to parietal, temporal, and then frontal lobes sequentially. Such a pattern is found in approximately 80% of cases; therefore, MRIs strongly suggest the diagnosis of X-ALD.

**Zellweger syndrome**

Zellweger or cerebrohepatorenal syndrome is a rare neurometabolic disorder characterized by areduction or absence of functional peroxisomes in cells. 

Zellweger spectrum disorders include three peroxisome biogenesis disorders as follows: Zellweger syndrome, neonatal adrenoleukodystrophy, (NALD), and infantile Refsum disease.

Although all have a similar molecular basis, Zellweger syndrome is the most severe of the three disorders.

Infants with Zellweger syndrome show elevated very long chain fatty acids in their blood plasma. Cultured skin fibroblasts obtained from patients show elevated very long chain fatty acids, phytanic acid,and plasminogen. 

Neurological manifestations defined as neuronal migrational defects, craniofacial abnormalities, eye defects, chondrodysplasia punctata, and hepatomegaly. 

Craniofacial abnormalities include high forehead, epicanthal fold, large fontanel, and midface hypoplasia (17).

**Neuronal Ceroid-lipofuscinosis**

Neuronal Ceroid-lipofuscinosis (NCL) is a group of neurodegenerative diseases that represent a common inherited neurodegenerative disease in childhood. 

NCL has different types as follows: infantile, late infantile, juvenile, and adult types with juvenile type the most common. Various types of NCL are inherited fromautosomal recessive inheritance.

Several mutations have identified that deletion as the CLN3 gene as the most common mutation for the most common type (Juvenile type or Batten disease). The CLN1 mutation may give rise to the infantile or juvenile forms (18, 19).

Electroretinograms (ERGs) from these patients show electronegative electroretinogram with a severe loss of b waves and the histopathologic studies have revealed reduced all cell numbers from all retinal layers.

Seizures are a common sign that is often refractory to anti-epileptic drugs and electroencephalograms show that these patients’ have generalized atypical spike and slow wave complexes.

There is no current specific treatment for neuronal ceroid-lipofuscinosis.Therefore, the management of patients focuses on the control of seizures.

**Storage disease**

**Lysosomal storage disorders**

This group of storage diseases is a clinically heterogeneous group of neurometabolic disorders that neurological manifestations are prominent clinical findings.

Lysosome facilitates the degradation of various products of cellular turn over that are mainly derived from lysosomes through endocytosis.

Alternative pathways for substrate entry into lysosomes are phagocytosis.

Mucopolysaccharidoses comes from abnormalities in the turnover of keratin sulfate, heparin, and dermatan sulfate; Gaucher disease is due to glucocerebrosidase deficiencies; the accumulation of its substrate glycosphingolipid glucosylceramide; and Fabry disease results from alpha-galactosyl sphingolipids oligosaccharides.

Other lysosomal disorders are cystinosis and sialic acid storage disorders that involve a dysfunction of the intracellular membrane transport that mediates movement of the products outside the lysosome for the final step of excretion.

GM1 gangliosidosis is the results of a beta-galactosidase deficiency and the accumulation of ceramides caused by an acid ceramidase deficiency that causes Farber disease. 

GM2 gangliosidosis as Tay-Sachs disease (an alpha subunit of beta hexosaminidase deficiency); Sandhoff disease (a beta subunit of beta-hexosaminidase deficiency); and the GM2 AB variant are other types of lysosomal storage diseases. 

The complex lipid of gangliosides is found predominantly in gray matter in the brain. Classic forms of gangliosidoses present in early and late infancy and are usually fatal during these periods.

In the early infantile form of GM1, gangliosidoses dysmorphic features may present at birth and often hepatosplenomegaly is noted. The bone deformity (dysostosis multiplex) is similar to mucopolysaccharidosis. Macular cherry red spot (CRS) is found in about 50% of patients and hypotonia, feeding difficulties, and failure to thrive may be evident in the first weeks and months of life. Affected infants develop spasticity, tonic spasms, and pyramidal signs; and infants who survive beyond 12 months exhibit decerebrate rigidity.

CRS is also a hallmark of Tay-Sachs disease. In the classic form of Tay-Sachs disease, affected infants have psychomotor deterioration and hyperacusis together with axial hypotonia. The size of head increases markedly and patients become spastic in the second year of life. 

Karimzadeh et al indicated that, for18 patients with GM2- Gangliosidosis (9 patients with Sandhoff and 9 with Tay Sachs disease), CRS was reported in 88% of patients. Also in this study, 7patients had macrocephaly, 3 had microcephaly, and 8 had a normal head circumference. Therefore, macrocephaly was not considered significant in this assessment (20).

The late onset form of the disease can manifest in childhood and adolescence with cerebellar ataxia, and dysarthria due to cerebellar atrophy. Tonic clonic seizures and psychiatric disturbances are present and may be the initial problems. Peripheral neuropathy has been described and has been considered for late onset Tay-Sachs disease.

Sphingomyelinase deficiency causes an accumulation of sphingomyelin in Niemann-Pike type A, Arylsulfatase deficiency leads to accumulation of sulfatides in metachromatic leukodystrophy and galactocerebrosidase deficiency causes Krabbe disease. 

There is wide heterogeneity in the clinical presentation of these diseases. Clinical manifestation may be evident prenatally or at any time from birth to adulthood. For example, Gausher disease and Fabry may not be associated with prominent symptoms during infancy and childhood.

Type 1 Gausher disease as the most common lysosomal storage disease presents with hematologic and visceral findings. Type2 presents with the acute neuropathic form during infancy that leads to spasticity, dysphagia, and death before 2–3 years of age. 

Type 3 Gausher disease that manifests before 2 years of age shows chronic neurological problems including seizures, ataxia, and extrapyramidal rigidity. 

Oculomotor apraxia (especially horizontal saccadic eye movement failure) is a characteristic finding in neuropathic Gausher disease. Supra nuclear horizontal gaze palsy may be the single neurological problem in Gausher disease type 3.

There are at least three disorders as follows: Fabry, Danon, and Hunter syndrome (MPS II) that are transmitted as X-linked traits but most lysosomal storage diseases are transmitted in autosomal recessive inheritance Pinto et al reported a combined prevalence of about 1 in 5000–8000 for lysosomal storage diseases. 

Some defined ethnic groups such as the Ashkenazi population has a high frequency of some lysosomal storage diseases like Tay-Sachs, Gausher, and Niemann- Pick type A.

Enzyme replacement therapy for Gausher disease, Fabry, Pompe (Glycogen storage disease type II), Hurler-Scheie (MPS I H/S), Hunter, and Maroteaux- Lamy disease (MPS VI), is available.

Niemann-Pick type C disease is a rare progressive neurodegenerative disorder with autosomal recessive inheritance. The underlying biochemical defect is an impaired lipid metabolism, which is caused by pathological mutations of either the NPC1 (95%) or the NPC2 (5%) gene.

Niemann-Pick disease type C (NPC) has wide clinical heterogeneity and can present during the neonatal period, infancy, childhood, or adulthood. Pre- or peri-natal and early-infantile diseases are usually detected from prolonged neonatal jaundice and hepatosplenomegaly. 

In addition, very young patients often display central hypotonia and developmental delays (21,22,23).

Late infancy and juvenile Niemann-Pick disease type C can present as dystonia, dysarthria, or dysphagia, and, usually presents with ataxia and supranuclear gaze palsy. Patients from these two groups often display gelastic cataplexy, epilepsy, and cognitive impairment that can manifest as learning disabilities (24).

Substrate reduction therapy with miglustat has been reported, which can be a stabilizer Neurological manifestations in patients with Niemann- Pick disease type C; Study of Karimzadeh and her colleagues on 21 Iranian NPC Patients supported previous findings demonstrating stabilization of neurological disease with miglustat in children diagnosed in the early stages of the disease. 

Similar to published previous reports, these patients did not show improvement in systemic manifestations. 

Despite the other studies about gastrointestinal problems (diarrhea and anorexia) during Miglustat therapy that they had to discontinue treatment due to adverse effects, Iranian patients tolerated well the Miglustat (25).

**Urea cycle disease**

Urea cycle enzyme disorders include carbamylphosphate synthetase deficiency,arginosuccinic aciduria, ornithine transcarbamylase deficiency, citrullinemia, arginase deficiency, and N-acetylglutamate synthetase deficiency.

Deficiencies of enzymes in Urea cycle disorders lead to hyperammonemia and present classically with a loss of consciousness and coma.

The most common type of presentations is ornithine transcarbamylase deficiency. The inheritance of this disorder is X-linked and presents in males.

In Urea, cycle disease hyper-ammonia is not associated with acidosis as it is associated with acidosis in organicacidemia. 

Patients with Urea cycle disease, are more likely to have respiratory alkalosis. Orotic aciduria is found in patients with ornithine transcarbamylase deficiency. Orotic aciduria also is found in citrullinemia and argininemia. In patients without orotic aciduria, the expected diagnosis is carbamyl phosphate synthetize deficiency or N-acetyl glutamate synthetize deficiency.

In the acute hyperammonemia all intake of protein or other sources of nitrogen should be stopped. Glucose is used for the promotion of hyper-anabolism, water, and electrolytes are provided intravenously. Arginine to keep urea cycle supplied and intravenous sodium benzoate and sodium phenyl acetate are used for excretion of waste nitrogen. 

Hemodialysis is often required in acutely hyperammonemic newborns.

Follow-ups of children who treated for pharmacologic therapy showed up to a 90% survival rate but 80% of these patients had significant developmental disabilities.

In addition, a significant relationship was found in these patients between the duration of neonatal hyperammonemic coma, the level of mental decline, and IQ.

**Neuroimaging findings in neurometabolic disorders**

MRIs are an important neuroimaging tool for evaluating neurodevelopmental disorders.

Routine MRIs are usually the first step in the evaluation of neurodevelopmental and neurometabolic disorders.

MRIs are the choice for neuro-image studies of brain structural abnormality, normal and abnormal myelination, and functioning.

Today, functional MRIs have an important value for motor and learning abilitiesof the brain. 

MRS is a reliable and noninvasive method to study the brain biochemistry in neurometabolic disorders.

MRS shows the regional metabolic abnormality as an etiologic factor of the disease.In addition, we can use MRS to evaluate the rapeutic management and progression of neurometabolic diseases.

**MRS for studying of neurometabolic disorders**

The analysis of metabolic peaks in MRS in combination of clinical finding (History & physical examination), metabolic study, and biochemical data are a good method for confirmation of neurometabolic disease.

MRS is a good predictor for response to treatment, disease severity, and outcome of disorders.

Proton MR Spectroscopy (PMRS) is a diagnostic test for specific neurometabolic disorders, for example peak of N- acetylaspartate (NAA) in Canavan disease due to aspartoacylase deficiency, peak of Phe. in phenylketonuria (PKU) due to phenylalanine hydroxylase

deficiencies, peak of leucine, isoleucine,and valine in MSUD disease due to Debranche enzyme deficiency and also is diagnostic for Urea cycle disorders. 

MRS in phenylketonuria shows a peak of phenylalanine at 7.37 ppm. The size of the peak is useful for monitoring of serum phenylalanine and monitoring of treatment. 

MRS in Maple Syrup Urine disease shows macromolecule peaks typically seen at 0.9 ppm to represent branched chain amino acids and branched chain alpha ketoacids. 

MRS reveals the peak of a lactate doublet at 1.33 ppm in mitochondrial disorders. MRS in mitochondrial encephalopathy with lactic acidosis (MELAS) and mitochondrial with ragged red fiber (MERRF)shows lactate rising.In Kerns-Sayre syndrome, MRS for affected regions in the brain show increased lactate and reduced NAA (26).

Additionally,proton MR spectrum (TE=288) in Leigh disease shows a large lactate doublet at 1.33 ppm in the affected regions (basal ganglia, brain stem, and white matter). In these patients, NAA peaks in Proton MR Spectrum (TE=144) are markedly reduced in size.

**Fig 5:**
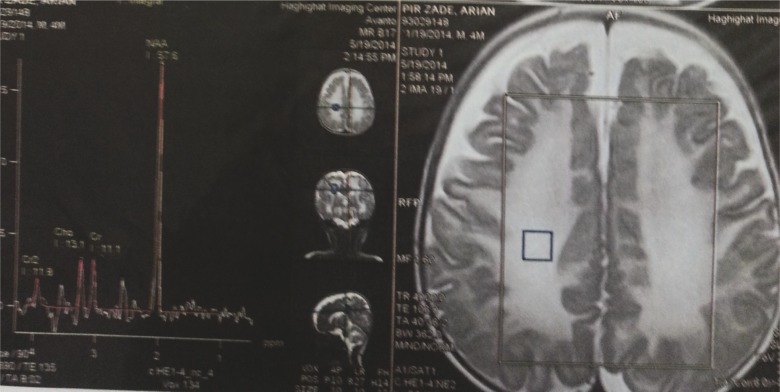
MRS in a patient with Canavan disease shows a markedly increased level of N-acetylaspartic acid (NAA)

In some studies, MRS revealed, even in carriers of amino acid disorders, slightly higher levels of brain amino acids were detected. Weglage and coworkers measured levels of brain phenylalanine by MRS and found that in carriers a PKU level of phenylalanine was higher than for non-carriers.

During episodes of acute metabolic catabolism in organicacademia and Maple syrup disease accumulations of Lactate and ketoacids have been detected. 

In a creatine deficiency, H proton MRS, demonstrates severely decreased or absent for creatine/ phosphocreatine peak. In addition, MRS can be used for monitoring treatment with creatine monohydrate in these patients.

On the other hand, H-MRS is used for early detection of asymptomatic high-risk children for neurometabolic disease.

MRS can be used for monitoring the response to treatment.

In some studies, MRS has been used for monitoring the response of dichloroacetate and other therapeutic agents in mitochondrial group diseases.

**Fig 6:**
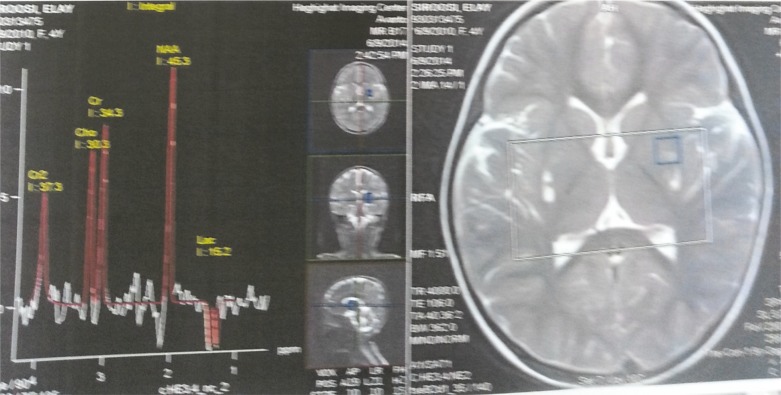
Cranial MRI and MRS in a patient with glutaric aciduria type I and Axial MRI shows an abnormal signal in bilateral basal ganglia with bilateral prominent sylvian fissures

**Diffusion tensor imaging in neurometabolic disorders**

Diffusion tensor imaging has been used for the evaluation of white matter disorders.

This modality is a noninvasive evaluation for diagnosis of neurodegenerative disorders involving predominantly white matter and possible leukodystrophies.

In X-linked adrenoleukodystrophy, white matter demyelination typically begins in posterior white matter (occipital region) and then extends anteriorly.

In addition, diffusion tensor imaging has been used for the evaluation of water diffusion in mitochondrial disorders.

Diffusion tensor imaging is a helpful modality for study of neurometabolic disorders involving white matter structures such as phenylketonuria and ornithine transcarbamylase deficiency.

**Epilepsy and EEG patterns for neurometabolic diseases**

Although, Neurometabolic disorders are not a frequent cause of epilepsy, epilepsy is a frequent symptom of neurometabolic disease. Epilepsy in this group of disease has different types of seizures and EEG patterns. 

Epilepsy in neurometabolic disorders can be a prominent symptom especially in newborns and early infants. 

In these patients, epilepsy can cause neurodevelopmental regression in motor, mental, and speech milestones. It is often referred to as epileptic encephalopathy. 

The type of epilepsy depends on the age rather than the type of neurometabolic disorders. The common types of epilepsy in these patients are early myoclonic infantile spasm, and west syndrome in infancy; generalized tonic clonic seizures in late infantile and childhood period; and unexplained status epilepticus is common in these patients (27,28).

The history of these patients also shows consanguinity marriage in parents, positive family history of similar cases, neurodevelopmental delays, psychomotor regression, and abnormal neurological examination.

EEGs are abnormal in most of neurometabolic epileptic patients.

**Fig 7:**
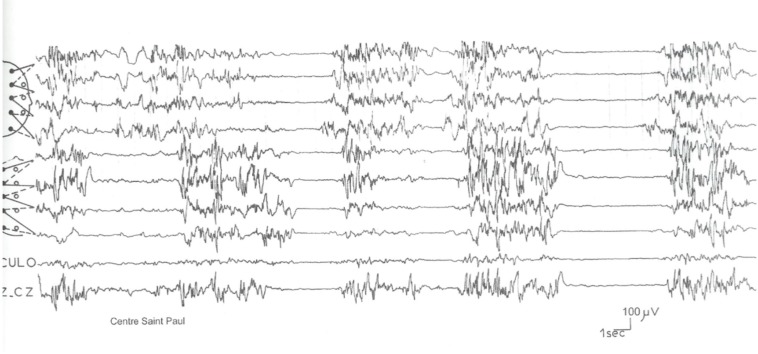
Burst suppression patterns in a 4.5-month-old infant with early myoclonic encephalopathy

Burst suppression patterns in EEG are often due to neurometabolic diseases, especially in non-ketotic hyperglycinemia, urea cycle disorders, organic aciduria, sulfite oxidase deficiency, and biotinidase deficiency in newborn and early infants (Figure 7).

**Fig 8:**
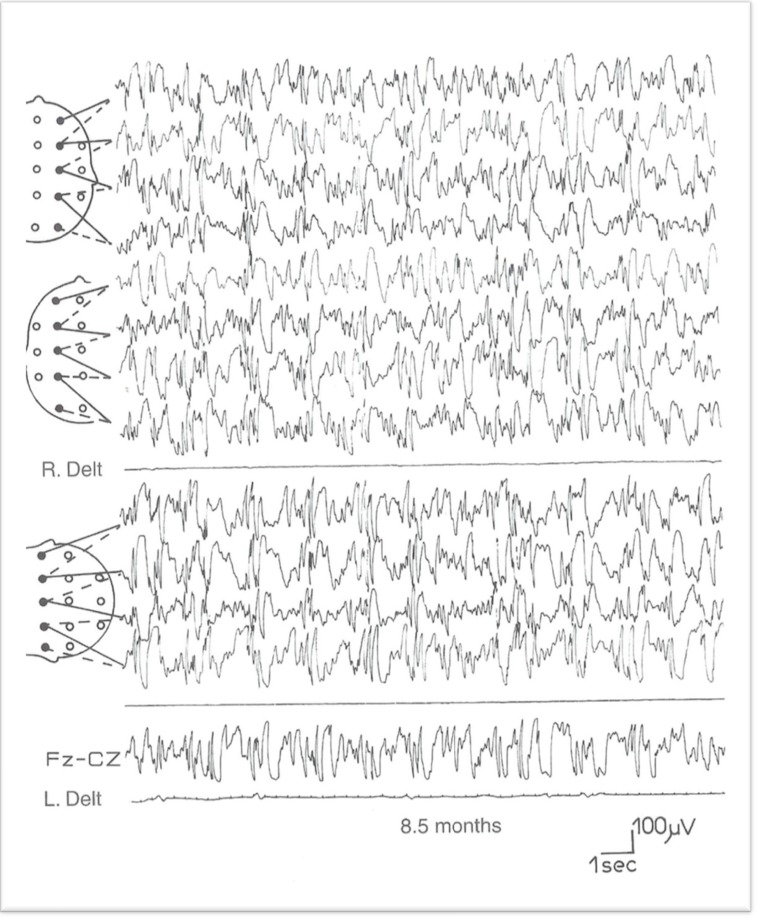
Hypsarrhythmia patterns with mixed asynchronous slow waves and spike waves in an 8.5-month-old infant with West syndrome

EEGs in West syndrome show a hypsarrhythmia pattern. It is defined as a chaotic pattern and irregularity throughout tracing (Figure 8).

In childhood, neurometabolic-causing epilepsy presents as progressive myoclonic epilepsy.

**Fig 9:**
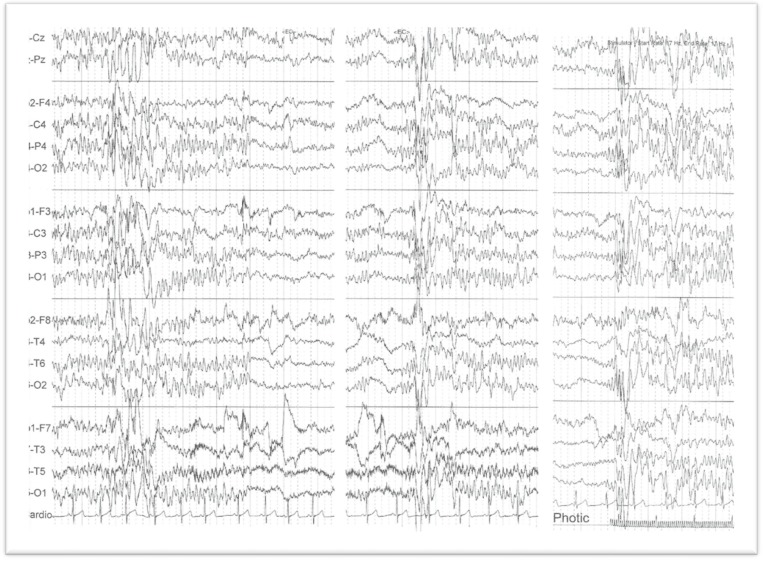
Slow spike waves with fast spike components in a 13-year-old boy with Unverricht-Lundborg Syndrome

The main cause of progressive myoclonic epilepsy (PME) is neuronal ceroid-lipofuscinosis (NCL), other causes of PME are MERRF (myoclonic epilepsy ragged ref fiber), sialidosis, Lafora disease, Gaucher disease type III, GM2 gangliosidosis, and Unverricht-Lundborg disease (Figure 9).

Rhythmic delta waves associated with paroxysmal response during photic stimulation in the EEG are highly suggestive of PME.

Epilepsy of neurometabolic disease is often resistance to antiepileptic drugs. When an early infant shows refractory seizures, neurometabolic disease should be considered.

In some of neurometabolic disease with refractory to antiepileptic drugs, specific treatment of basic neurometabolic diseases in these patients such as supplementation cofactors, diet therapy, and/or specific treatment of deficient metabolites can help control seizures.

For example, refractory seizures in biotinidase deficiency have a good response to high dose of biotin. Some antiepileptic drugs may worsen neurological and clinical conditions of patients with neurometabolic disorders such as valproic acid in mitochondrial disease; urea cycle disease; and organic-academia cause seizure worsening, loss of consciousness, and abnormal liver functioning tests.
